# Uncovering antiobesity-related hypertension targets and mechanisms of metformin, an antidiabetic medication

**DOI:** 10.1080/21655979.2021.1954581

**Published:** 2021-08-02

**Authors:** Lu Yang, Jianxin Yang, Xiao Liang, Wenjun Huang, Xiaoxi Zhang, Rong Li

**Affiliations:** a Faculty of Basic Medicine, Guilin Medical University, Guilin, PR China; b Cardiology Department Area 1, Guigang City People’s Hospital, the Eighth Affiliated Hospital of Guangxi Medical University, Guigang, Guangxi, PR China; c Laboratory of Environmental Pollutants and Integrative Omics, Guilin Medical University, Guilin, PR China

**Keywords:** Metformin, obesity, hypertension, bioinformatics findings

## Abstract

Metformin, a common clinical drug used to treat diabetes mellitus, is found with potential antiobese actions as reported in increasing evidences. However, the detailed mechanisms of metformin-antiobesity-related hypertension remain unrevealed. We have utilized the bioinformatics strategy, including network pharmacology and molecular docking analyses, to uncover pharmacological targets and molecular pathways of bioactive compounds against clinical disorders, such as cancers, coronavirus disease 2019. In this report, the *in-silico* approaches using network pharmacology and molecular docking was utilized to identify the core targets, pharmacological functions and mechanisms of metformin against obesity-related hypertension. The networking analysis identified 154 differentially expressed genes of obesity and hypertension, and 21 interaction genes, 6 core genes of metformin treating obesity-related hypertension. As results, molecular docking findings indicated the binding capability of metformin with key proteins, including interleukin 6 (IL-6) and chemokine (C-C motif) Ligand 2 (CCL2) expressed in obesity- and hypertension-dependent tissues. Metformin-exerted antihypertension/obesity actions involved in metabolic regulation, inflammatory suppression. And antihypertension/obesity mechanisms of metformin were revealed, including regulation of inflammatory and immunological signaling pathways for ameliorating microenvironmental homeostasis in targeting tissues. In conclusion, our current bioinformatics findings have uncovered all pharmacological targets, biological functions and signaling pathways of metformin treating obesity-related hypertension, thus promoting its clinical application in future.

## GRAPHICAL ABSTRACT

**Figure:**
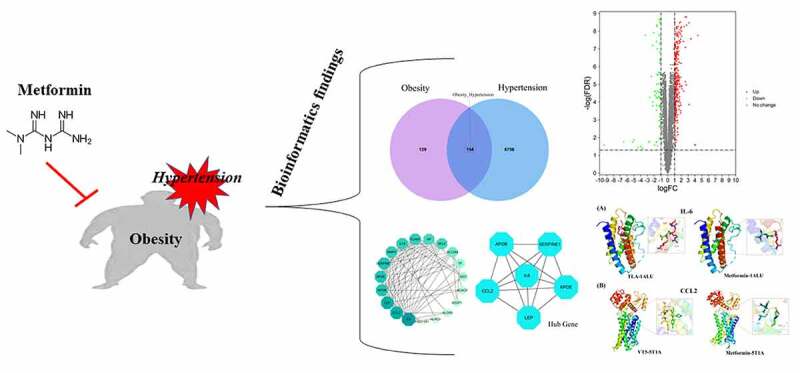

## 1. Introduction

Obesity, a possible health risk, may induce other chronic diseases over time, such as cardiovascular diseases, cancer, metabolic disorder, hypertension [1]. Reportedly, the onset of obesity is associated with interaction in genetic and environmental factors, changes in material metabolism and endocrine function, neuropsychiatric factor [2]. More obviously, the epidemiological data indicate that the prevalence of obesity is rising rapidly year by year, such as western countries [3]. In China, the prevalence of children or adult obesity is increasing in recent decade, and then certain plans issued by Chinese government aim to prevention and control of obesity [4]. Hypertension, also known as high blood pressure, is a common clinical disease that blood pressure characterizes with abnormal increment, such as 140/90 mm Hg or greater [5]. It is evident that hypertension is one of most important risk factors in cardiovascular and cerebrovascular diseases, causing increased disability and mortality [6]. The potentially effective management of hypertension includes alimentary control, lifestyle balance, and pharmacotherapy [7]. Clinically, the antihypertensive drug may prescribe with monotherapy or combined therapy [8]. Mounting data indicate that pathophysiological connection of hypertension and obesity is positively evidenced, such as insulin resistance, metabolic function changes, and hormonal alteration, and homeostasis dysregulation [9]. Therefore, the occurrence and prevalence of obesity-associated hypertension are high in the world, and hypertension and obesity have become a major health challenge in human being. Metformin, a common antidiabetic agent, has been reported with potent anti-cancer benefits via regulating multiple signaling pathways in cancer cells, including mammalian target of rapamycin (mTOR) signaling pathway [10]. More divertingly, numerous findings suggest that metformin may be used for obese control via suppressing obesity-induced low-grade inflammation and ameliorating macrophage function [11], and reconstructing gut microbiota [12]. In addition, metformin is found with antihypertensive actions based on clinical and experimental findings [13,14]. Taken together, metformin may be used for antihypertension and obesity on the basis of current review analysis. However, detailed antihypertension and obesity mechanisms of metformin remain unclear totally. In *in-silico* strategy, network pharmacology and molecular docking methods can be utilized to screen and identify all antidiseased core genes and mechanisms of bioactive compounds [15,16]. Intriguingly, our previous studies demonstrate that network pharmacology and molecular docking approaches/analyses are used for effective revelation of core genes and pharmacological mechanisms of plumbagin, vitamin C treating clinical disorders, including liver cancer, sepsis, pneumonia, and liver injury [17–20]. Overall, our current bioinformatics study was designed to identify all key genes of metformin for antiobesity-related hypertension via molecular docking analysis, as well to reveal the antiobesity-related hypertension actions, pharmacological pathways of metformin.

## 2. Methods

### 2.1. Screening of candidate gene in obesity and hypertension

Using the Gene Expression Omnibus (GEO) sub-database in National Center for Biotechnology Information (NCBI) database (https://www.ncbi.nlm.nih.gov/), we used ‘Obesity’ as searching keyword to obtain the data sets in gene expression profiling in patients, including GSE59034 and GSE112307. A total of 16 normal-weight adults and 16 obese patients were selected from GSE59034 data setting. Harvesting the output of before and after weight loss in adult obese patients, R-language software (https://www.r-project.org/) was used to perform gene chip quality analysis and to identify differentially expressed genes (DGEs). DGEs were identified via the screening conditions of | logFC |≥1 and adjusted *P* value < 0.05 by use of Limma package. The canonical genes of hypertension were screened out through Genecard, DisGeNET, Online Mendelian Inheritance in Man (OMIM) databases, and gene-functional module in National Center for Biotechnology Information (NCBI). Subsequently, the DGEs-associated obesity and the hypertension-related genes were mapped using Venn online tool to obtain all intersection genes in obesity and hypertension. All intersection genes were submitted to bioinformatics online tool to gain the up- and down-regulated differential genes for plotting Volcano map [21,22].

### 2.2. Metformin-associated gene screening and intersection gene identifying

The pharmacological genes of metformin were collected through the databases of Traditional Chinese Medicine Systems Pharmacology (TCMSP), Swiss Target Prediction, PharmMapper, Batman, Drugbank, and SuperPred. The genes of metformin were reviewed (Swiss-Prot in the Uniprot database) after Human target correction. Subsequently, the candidate genes of metformin and obesity-related hypertension were mapped for collection of all intersection targets in metformin treating obesity/hypertension through Venn diagram analysis [23,24].

### 2.3. Hug target screening and protein–protein interaction (PPI) network constructing

After mapping, the antiobesity-related hypertension targets of metformin were obtained accordingly. And then the functional protein association network was produced using STRING databank, in which lowest interaction value was set to 0.09. And the data were imported into Cytoscape_v3.6.1 software to construct a PPI network of metformin-antiobesity/hypertension targets. In details, Network Analyzer in Cytoscape software was used to determine the topological parameters, including the median and maximum degrees of freedom. On the basis of Degree Value (DV), the upper limit of screening range was the maximum DV in topology data, and the lower limit was the median degree of freedom for identifying hug targets [25,26].

### 2.4. Enrichment analysis and integrative network construction

By using Database for Annotation,Visualization and Integrated Discovery (DAVID) database, the functionally biological process and Kyoto Encyclopedia of Genes and Genomes (KEGG) pathway from all hug targets were revealed. In addition, the R packages, such as ‘GOplot,’ were used to harvest and visualize ontology (GO)-based biological processes and signaling pathways, as well to plot the bubble chart, Circos map, and histogram. Furthermore, construction of visual graphics in biological processes and molecular pathways was completed via network analysis using Cytoscape software for visualization of metformin treating obesity and hypertension [27,28].

### 2.5. Molecular docking determination

As described in our previous reports [29,30], the three-dimensional (3D) structure of metformin-related compound was obtained from PubChem database. The protein structure of hug targets of obesity-related hypertension was gained from Protein Data Bank (PDB) database. The 3D structure of metformin was downloaded from ChemBio3D Draw setting in Chem Bio Office 2010 software to optimize the molecular mechanics 2 (MM2) force field. The pdbqt-structural file for virtual screening was processed through Raccoon software. The protein, hydrogenation, Gasteiger charge, and nonpolar hydrogen were processed via MGLTools (1.5.6) in Autodock Vina software. The original pdb file format was converted to pdbqt file format using Autodock Vina prior to docking biologically. The active center by use of docking was identified through Grid Box functional setting, including all surrounding residues centered on original ligand. The rationality of docking parameter settings was normalized through root mean square deviation between docked ligand molecule and original ligand molecule.

## 3. Results

### 3.1. The identified genes of obesity and hypertension

**Figure 1.**
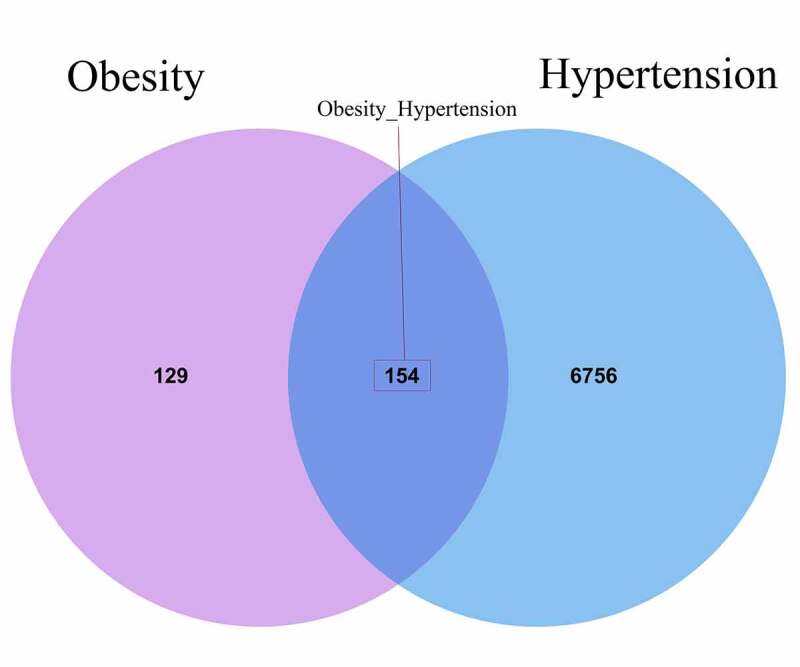
A Venn diagram showed all pathological genes both in obesity and hypertension, and total 154 interaction genes were identified accordingly

**Figure 2.**
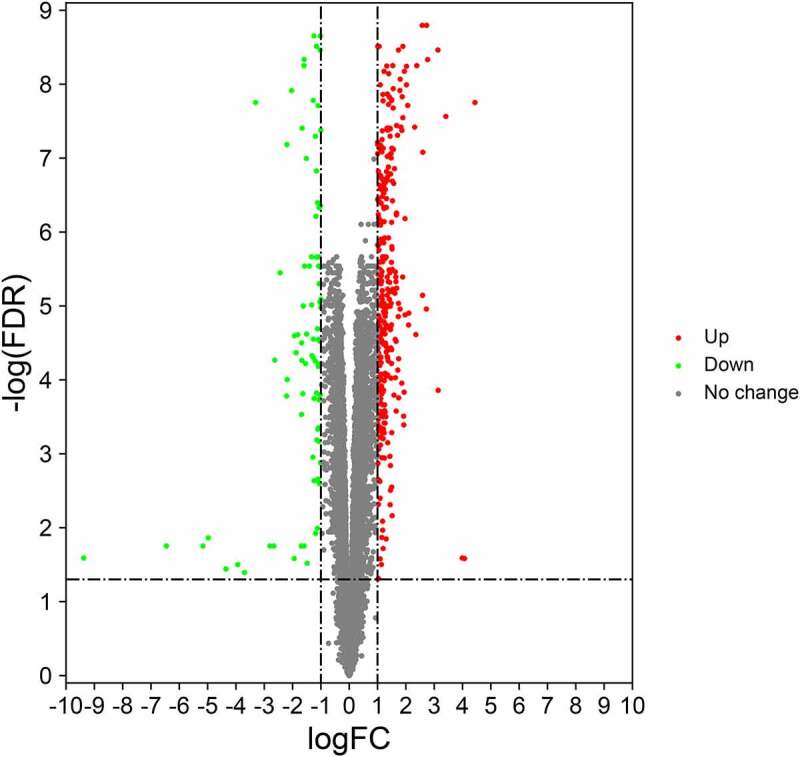
A Volcano map was plotted to feature the differentially expressed genes of obesity and hypertension

After being processed via Gene Expression Omnibus database, a total of 283 differential genes related to obesity were screened out. And other 6910 genes associated with hypertension were collected accordingly. And then 154 intersection genes of obesity and hypertension were identified in Venn diagram, as shown in Figure 1. For further bioinformatics analysis, all DGEs in obesity and hypertension were identified in Volcano map, including 104 upregulated and 50 downregulated genes (Figure 2). More detailed information was displayed in Supplementary file 1.

### 3.2. The pharmacology and intersection genes of metformin and obesity-related hypertension

**Figure 3.**
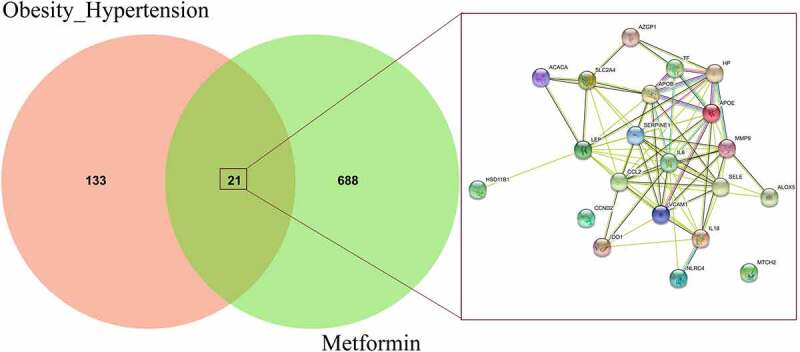
Further Venn diagram analysis highlighted all 21 interaction genes of metformin and obesity-related hypertension before networking visualization

By using different databanks for screening and identifying pharmacological targets, a number of 709 genes of metformin were harvested after rectification. As a result, total 21 intersection genes of metformin and obesity-related hypertension were identified in Venn diagram, and all these genes were correlated in networking map (Figure 3).

### 3.3. The hug genes of metformin treating obesity-related hypertension

**Figure 4.**
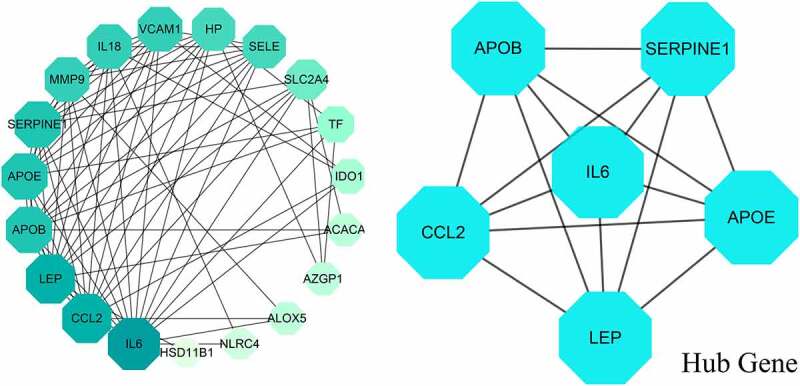
All 21 interaction genes of metformin and obesity-related hypertension were reciprocally connected in network map, and 6 hug genes of metformin against hypertensive obesity were screened out and identified

After topological analysis with 21 intersection genes, the median degree of freedom in target network was 7.895, and the maximum degree of freedom was 15. Therefore, the hug target screening criterion range was set from 8 to 16. Using this algorithm, all hug targets of metformin against obesity-related hypertension were identified accordingly, including IL6, CCL2, Leptin (LEP), Apolipoprotein B (APOB), serine protease inhibitor clade E member 1 (SERPINE1), Apolipoprotein E (APOE) (Figure 4).

### 3.4. The enriched and integrated findings of metformin treating obesity-related hypertension

**Figure 5.**
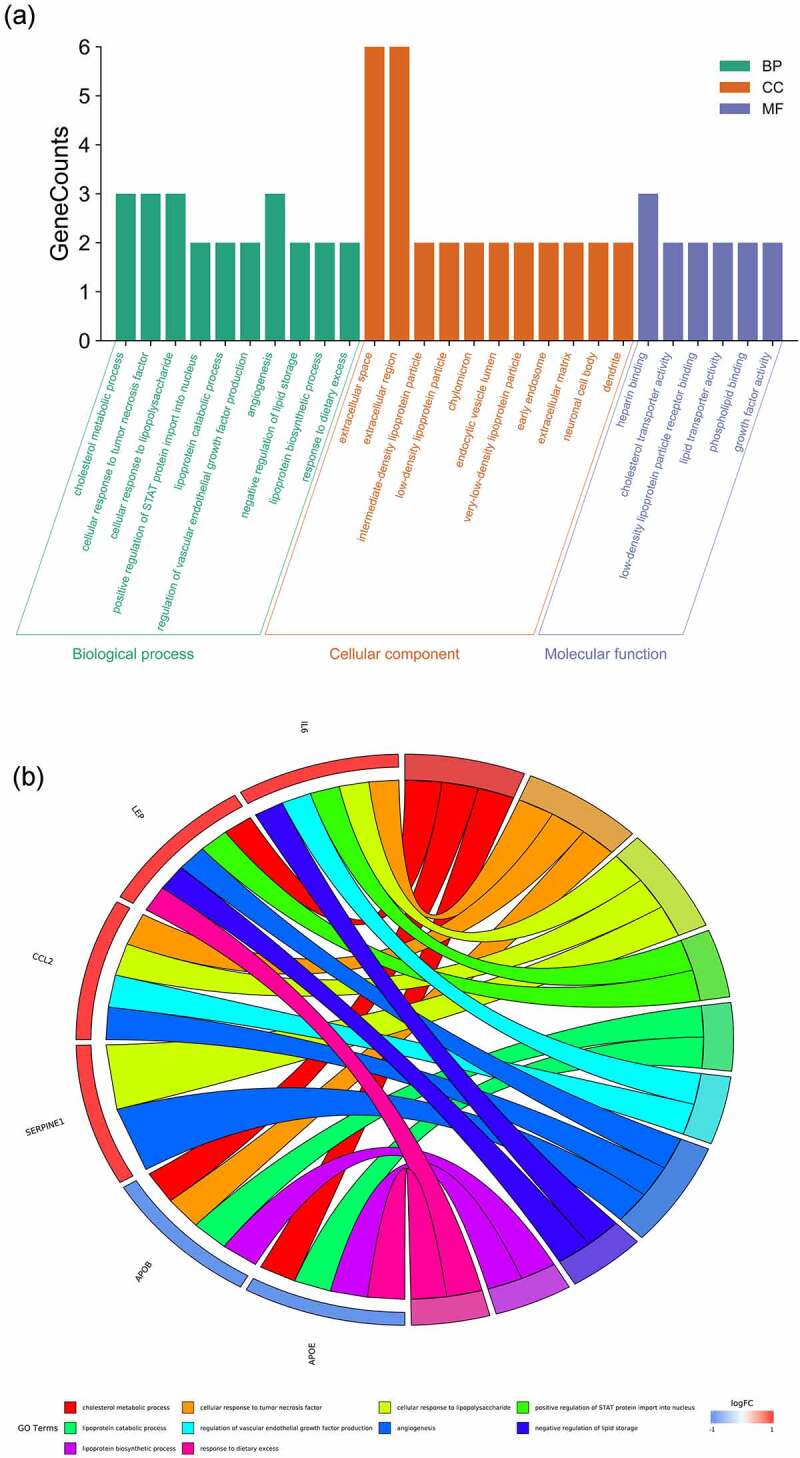
The biological functions from enriched analysis in hug genes were detailed, as highlighted in histogram (a) and circos diagram (b)

**Figure 6.**
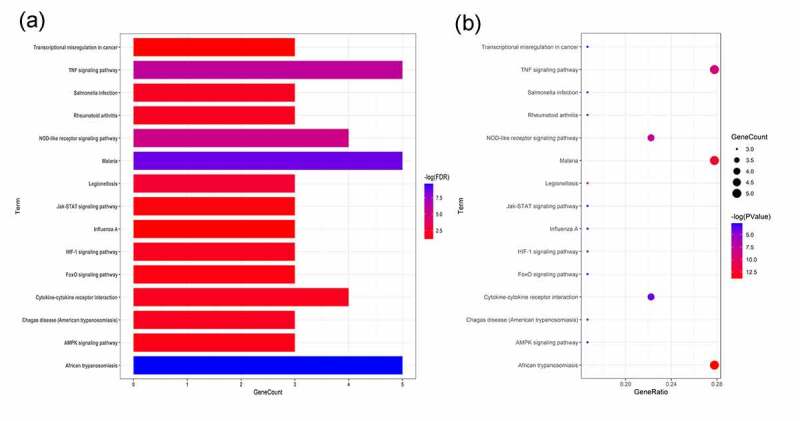
The KEGG-pathways associated molecular mechanisms using enriched analysis in hug genes were uncovered, as visualized in histogram (a) and bubble diagram (b)

**Figure 7.**
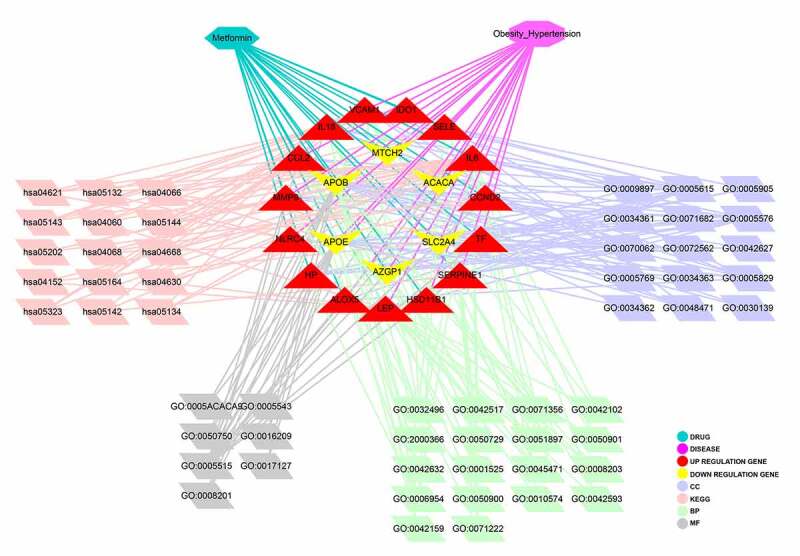
The integrated networking profiles of metformin-target-GO-KEGG-obesity/hypertension were characterized and visualized

The enrichment analyses of gene ontology (GO) and KEGG pathway using six hug genes were conducted through DAVID database and R language-related packages. The output data of GO- and KEGG-based enrichments of metformin treating obesity-related hypertension showed in histogram and Circos (Figure 5), bubble chart and histogram (Figure 6). These findings indicated that the biological processes associated with metformin treating obesity-related hypertension were mainly involved in cholesterol metabolic process, cellular response to tumor necrosis factor, cellular response to lipopolysaccharide, positive regulation of STAT protein import into nucleus, lipoprotein catabolic process, regulation of vascular endothelial growth factor production, angiogenesis, negative regulation of lipid storage, lipoprotein biosynthetic process, response to dietary excess (detailed in Supplementary file 2). The cell components in metformin treating obesity-related hypertension were basically related to extracellular space, extracellular region, intermediate-density lipoprotein particle, low-density lipoprotein particle, chylomicron, endocytic vesicle lumen, very-low-density lipoprotein particle, early endosome, extracellular matrix, neuronal cell body, dendrite (detailed in Supplementary file 3). The molecular functions of metformin treating obesity-related hypertension predominantly included heparin binding, cholesterol transporter activity, low-density lipoprotein particle receptor binding, lipid transporter activity, phospholipid binding, growth factor activity (detailed in Supplementary file 4). Moreover, 15 KEGG pathways of metformin treating obesity-related hypertension were revealed, including African trypanosomiasis, Malaria, TNF signaling pathway, NOD-like receptor signaling pathway, Legionellosis, Salmonella infection, Rheumatoid arthritis, Cytokine-cytokine receptor interaction, HIF-1 signaling pathway, Chagas disease (American trypanosomiasis), AMPK signaling pathway, FoxO signaling pathway, Influenza A, Jak-STAT signaling pathway, Transcriptional misregulation in cancer (detailed in Supplementary file 5). Furthermore, the network correlation with all bioinformatics findings of metformin against obesity/hypertension were featured and displayed in metformin-target-GO-KEGG-obesity/hypertension map (Figure 7).

### 3.5. The molecular docking data

**Figure 8.**
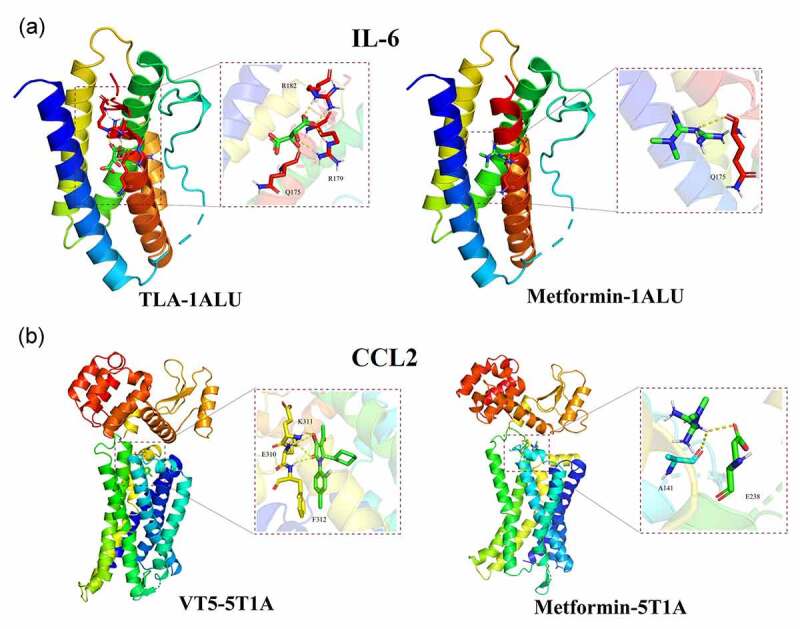
By use of molecular docking analysis, bio-structurally binding capability of metformin with target proteins in obesity/hypertension, including IL-6, CCL2, was identified and demonstrated

Using PDB database, IL-6 and CCL2 proteins in obesity and hypertension were screened out, and then 1ALU in IL-6 was selected for docking with metformin biologically. The active cavity box in 1ALU using parameter setting with center x-y-z was −7.646, −12.833, 0.083, and size x-y-z was 14, 14, and 10, respectively. And the RMSD of original ligand was 2.344 Å. The hydrogen bond between original ligand TLA and 1ALU protein were found with acting on three amino acid residues, including Q175 (2.8 Å), R179 (2.0 Å), and R182 (1.8 Å). Metformin were identified with interaction of amino acid residue Q175 (2.6 Å) via formed hydrogen bond (Figure 8(a)). In addition, 5T1A in CCL2 protein were used to bio-structurally dock with metformin. The active cavity box in 5T1A using parameter setting with center x-y-z was 5.632, 28.349, 187.638, and size x-y-z was 20, 24, and 20, respectively. And the RMSD of original ligand was 3.353 Å. The hydrogen bond between original ligand VT5 and 5T1A protein were found with acting on three amino acid residues, including F312 (2.1 Å), K311 (2.7 Å), and E310 (2.8 Å). Metformin were identified with interaction of amino acid residue E238. (2.4 Å) and A141 (2.8 Å) through formation of hydrogen bond (Figure 8(b)). All current *in-silico* findings might display the binding capability of metformin with IL-6, CCL2 proteins in obesity, and hypertension.

## 4. Discussion

Obesity may induce certain noninfectious diseases, such as diabetes mellitus, fatty liver. And the increased incidence of obesity is a rapidly evolving situation in developing countries, including China [31]. Hypertension, one of the complications induced by obesity, will cause organ damage over time if uncontrolled effectively [32]. However, clinical drug therapy against obesity combined with hypertension is prescribed limitedly. Metformin, an antidiabetic or hypoglycemic drug, is prescribed to treat obese diabetes in clinical practice [33]. Additionally, metformin is found as a promising medicine to reduce body weight in overweight or obese individuals [34]. In underlying anti-obese mechanism, metformin may benefit from normalizing the metabolic functions via regulation of gut microbiota for suppressing inflammatory reaction and enhancing immune response in obese mice [35]. In preliminary antihypertensive mechanism, metformin may contribute to remission of hypertension through activating sirtuin 3/AMP-activated protein kinase (SIRT3-AMPK) signaling pathway [36]. Although antiobesity/hypertension action of metformin is found, the detailed mechanisms are still unrevealed completely. As reported in our previous findings, in this study, network pharmacology and molecular docking analyses are used to uncover the antihypertension/obesity targets and mechanisms of metformin. As results, the identified findings using network pharmacology analysis had identified total six hug genes of metformin treating obesity/hypertension, including IL6, CCL2, LEP, APOB, SERPINE1, and APOE. In further bio-structural validation, *in-silico* data using molecular docking analysis had evidenced the biologically binding competency of metformin with core proteins within obesity and hypertension. And computational analysis suggested that metformin may effectively dock with 1ALU, 5T1A residues in IL-6, CCL2 proteins in obesity and hypertension. IL-6, an immunomodulator mainly produced by T cells, macrophages, or endothelial cells, is found with involvement of autoimmune disorder, bacterial infection and metabolic side action [37]. It is reported that IL-6 may affect the homeostasis of energy and glucose in obese mice through activating IL-6 trans-signaling in central nervous system [38], and thus IL-6 may be a potential therapeutic target in treatment of obesity [39]. Inflammation can function as an essential role in development of pulmonary arterial hypertension, including IL-6 [40]. Therefore, IL-6 may be a promising therapeutic target for pulmonary arterial hypertension via regulation of intracellular IL-6 signaling [41]. CCL2, a chemokine released by immune cells, can drive immunocytes in the organs of liver, muscle and adipose, promoting the occurrence and development of the inflammatory reaction [42]. A cross-sectional study suggests that CCL2 may be a diagnostic biomarker for assessing inflammation and physical fitness in children with obesity [43]. Taken with current bioinformatic findings, the key therapeutic targets of IL-6, CCL2 in metformin treating obesity/hypertension were identified through molecular docking validation. As revealed in GO-functional biological processes, metformin-exerted antiobesity/hypertension actions involved in cholesterol metabolic process, cellular response to lipopolysaccharide, lipoprotein catabolic process, negative regulation of lipid storage, lipoprotein biosynthetic process, response to dietary excess. In addition, the antiobesity/hypertension activities exerted by metformin may be related to regulation of heparin binding, cholesterol transporter activity, low-density lipoprotein particle receptor binding, lipid transporter activity, phospholipid binding, growth factor activity. In antiobesity/hypertension mechanisms played by metformin, the key molecular pathways, including TNF signaling pathway, NOD-like receptor signaling pathway, cytokine-cytokine receptor interaction, AMPK signaling pathway, FoxO signaling pathway, Jak-STAT signaling pathway, were revealed in details. Our current bioinformatics findings indicated that metformin might exert potent antihypertension and obesity actions through functionally regulating inflammatory and immunological signaling pathways for improving metabolic homeostasis in obesity-regulated tissues and ameliorating blood pressure-dependent microenvironment. However, as limitations in current report, the bioinformatics findings using *in-silico* analysis should be clinically validated in future study to identify the use of metformin in the treatment of obesity-related hypertension.

## 5. Conclusion

Altogether, these bioinformatics evidences from network pharmacology and molecular docking analyses have uncovered detailed core targets, pharmacological functions, and mechanisms of metformin treating obesity-related hypertension. Attractively, metformin, a conventional drug for antidiabetic management, may be a promising medicine to treat obesity combined with hypertension.

## Supplementary Material

Supplemental Material
